# Pathology and virology of natural highly pathogenic avian influenza H5N8 infection in wild Common buzzards (*Buteo buteo*)

**DOI:** 10.1038/s41598-022-04896-7

**Published:** 2022-01-18

**Authors:** Valentina Caliendo, Lonneke Leijten, Marco W. G. van de Bildt, Ron A. M. Fouchier, Jolianne M. Rijks, Thijs Kuiken

**Affiliations:** 1 Department of Viroscience, Erasmus Medical Center, 3015 GE Rotterdam, The Netherlands; 2 Dutch Wildlife Health Center, Utrecht University, 3584 CL Utrecht, The Netherlands

**Keywords:** Ecology, Immunology, Zoology, Ecology, Diseases, Pathogenesis

## Abstract

Highly pathogenic avian influenza (HPAI) in wild birds is a major emerging disease, and a cause of increased mortality during outbreaks. The Common buzzard (*Buteo buteo*) has a considerable chance of acquiring the infection and therefore may function as bio-sentinel for the presence of virus in wildlife. This study aimed to determine the virus distribution and associated pathological changes in the tissues of Common buzzards that died with HPAI H5 virus infection during the 2020–2021 epizootic. Eleven freshly dead, HPAI H5 virus-positive Common buzzards were necropsied. Based on RT-PCR, all birds were systemically infected with HPAI H5N8 virus, as viral RNA was detected in cloacal and pharyngeal swabs and in all 10 selected tissues of the birds, with mean Ct values per tissue ranging from 22 for heart to 32 for jejunum. Based on histology and immunohistochemistry, the most common virus-associated pathological changes were necrotizing encephalitis (9/11 birds) and necrotizing myocarditis (7/11 birds). The proventriculus of two birds showed virus-associated necrosis, indicating tropism of this virus for the digestive tract. Our advice is to collect at least a miniset of samples including brain, heart, liver, and spleen, as these tissues were positive both by RT-PCR and for virus-antigen-associated lesions.

## Introduction

Highly pathogenic avian influenza (HPAI) is a major emerging disease, and a cause of mass mortality in wild birds during outbreaks^1–15^. The 2020–2021 epizootic of HPAI H5 virus in Europe was the biggest on record in both wild birds and poultry. Based on the animal disease notification system of the affected countries, over 1000 detections of HPAI H5 virus were reported in wild birds^4^. The subtype H5N8 was the first and most widespread genotype circulating during the first months of the epizootic (i.e., October–November 2020)^3,4^. Nevertheless, the virus reassorted multiple times, so that sixteen distinct genotypes co-circulated. Early surveillance showed that the first incursions in Europe of the HPAI H5N8 virus occurred in the fall of 2020; however, the epizootic season extended into winter–spring–summer of 2021, with the outbreaks mainly clustered in two peaks: in November 2020 and March 2021^4^. The highest number of detections in wild birds occurred in waterfowl species (order Anseriformes). The second highest number of detections occurred in raptors (orders Accipitriformes, Strigiformes, and Falconiformes); nine different raptor species were found positive, and Common buzzard (*Buteo buteo*, order Accipitriformes) accounted for the highest number of detections in raptors^4^. These birds are infected predominantly by ingesting infected prey. Medium-sized raptor species like the Common buzzard, that can hunt and feed on sizeable birds and ingest a high quantity of infected meat, are considered to be at high risk of becoming infected with HPAIV, and to die of related disease^10,11^. In this context, the investigation of Common buzzard mortality for HPAI virus could be used in addition to waterfowl mortality as a passive-surveillance system for early warning and duration of the presence of the virus in wild bird populations.

Wild raptors also have been affected by severe HPAI disease during earlier HPAIV epizootics^7–12,15^. Studies on those birds showed that HPAI viruses in wild raptors are primarily neurotropic, and that infected raptors usually present with neurologic signs due to encephalitis, often resulting in death^8,10,15^. The condition of some of the carcasses in those reports was suboptimal due to a combination of autolysis, scavenging and freezing, all factors that may impede detailed pathological assessment, in particular identification of specific cell types and evaluation of autolysis-prone tissues such as enteric epithelium.

Despite the high number of reported infected Common buzzards, we are not aware of any studies on the pathogenesis of infection with recent HPAI H5 viruses in this species. This is particularly relevant because, since the 2005–2006 HPAI H5N1 outbreak, HPAI H5 viruses have evolved rapidly and currently are thought to persist longer in wild bird populations^1–6^. Updated knowledge of the virology and pathology of infections with more recently circulating viruses is necessary in order to better understand the current pathogenesis of HPAI in Common buzzards; to provide updated guidance regarding the key tissues to collect for HPAI diagnosis at autopsies; and to compare the presence of virus in cloacal and pharyngeal swabs, routinely tested for HPAIV surveillance, with the presence of virus in the main tissues.

To determine the virus distribution and associated pathological changes in the tissues of Common buzzards that died with HPAI H5 virus infection in the Netherlands during the 2020–2021 HPAI epizootic, a collaboration was established among citizen scientists, animal organizations and researchers, aiming to retrieve and examine freshly dead Common buzzards. Eleven Common buzzards were examined and necropsied. All the birds tested positive for HP H5N8. This study documents the virological and pathological findings associated with 2020–2021 HPAI H5N8 infection in Common buzzards and discusses its implications.

## Results

### Serology

All birds tested negative for antibodies against avian influenza virus NP.

### Virology

Pharyngeal and cloacal swabs were positive for HPAI H5N8 virus by RT-PCR, with comparable viral RNA levels in pharyngeal and cloacal swabs (Table 1). Sequencing and molecular characterization of HPAI H5N8 virus, detected in the pharyngeal swab of Common buzzard B9, is publicly accessible (http://www.gisaid.com, A/Common Buzzard/Netherlands/4/2020, isolate EPI_ISL_1575129). Based on virus culture in MDCK-cell culture, three birds in total tested positive in pharyngeal (B9 and B11), and cloacal (B3 and B9) swabs.

**Table 1:** RT-PCR of the influenza A virus matrix gene-fragment results and virus titers in swabs and organs of H5N8-infected wild Common buzzards (*Buteo buteo*). *Cycle threshold (Ct) value, cut-off value is 40; ** all titers are given in TCID_50_ log 10; # this value is likely an outlier; n, negative (< 0.5 for virus titers); np, not present.

RT-PCR values* (virus titers)**											
Samples	Common buzzards										
	B1	B2	B3	B4	B5	B6	B7	B8	B9	B10	B11
Pharyngeal swab	28 (n)	30 (n)	28 (n)	33 (n)	23 (n)	32 (n)	31 (n)	36 (n)	20 (4.8)	29 (n)	25 (4)
Cloacal swab	32 (n)	27 (n)	26 (1.8)	24 (n)	27 (n)	33 (n)	23 (n)	26 (n)	22 (2.8)	25 (n)	21 (n)
Lung	np	32	25	25	25	32	31	31	29	20	17
Air sac	31	25	np	24	29	34	30	14	19	22	19
Liver	35	np	34	24	25	31	np	29	20	28	23
Spleen	35	24	31	30	31	32	31	29	25	28	21
Heart	24	16	23	31	28	32	31	16	22	18	11#
Kidney	37	np	np	29	26	31	34	26	26	30	20
Jejunum	34	np	32	np	27	35	34	26	21	35	20
Colon	np	34	33	30	31	27	34	30	19	28	23
Pancreas	30	34	np	26	24	36	35	29	20	32	32
Brain	25	32	21	25	32	31	34	24	24	26	18

All tissues tested were virus-positive by RT-PCR (Table 1), indicating a systemic spread of the infection (n.b., Ct values give an indication of the viral RNA amount in each tissue). Although their Ct values were relatively close together, the tissues with the higher content of viral RNA were, in descending order: heart, brain, air sac, spleen, and lung; the tissues with the lower content of viral RNA were, in descending order: liver, pancreas, colon, kidney, jejunum.

### Gross pathology

Birds did not present external lesions, and were in moderate to good state of nutrition. The crop of the birds was empty (an indication that they had not fed at least for 24 h prior death), and four birds had scarce food remains from a recent meat meal in the ventriculus. At internal examination only two birds presented gross abnormalities. When present, the main lesions were multifocal, well-delimited hemorrhages in the heart and in the brain of one bird. The liver of a different bird appeared swollen and with rounded margins. The other organs appeared grossly normal.

### Histopathology and Immunohistochemistry

**Figure 1:**
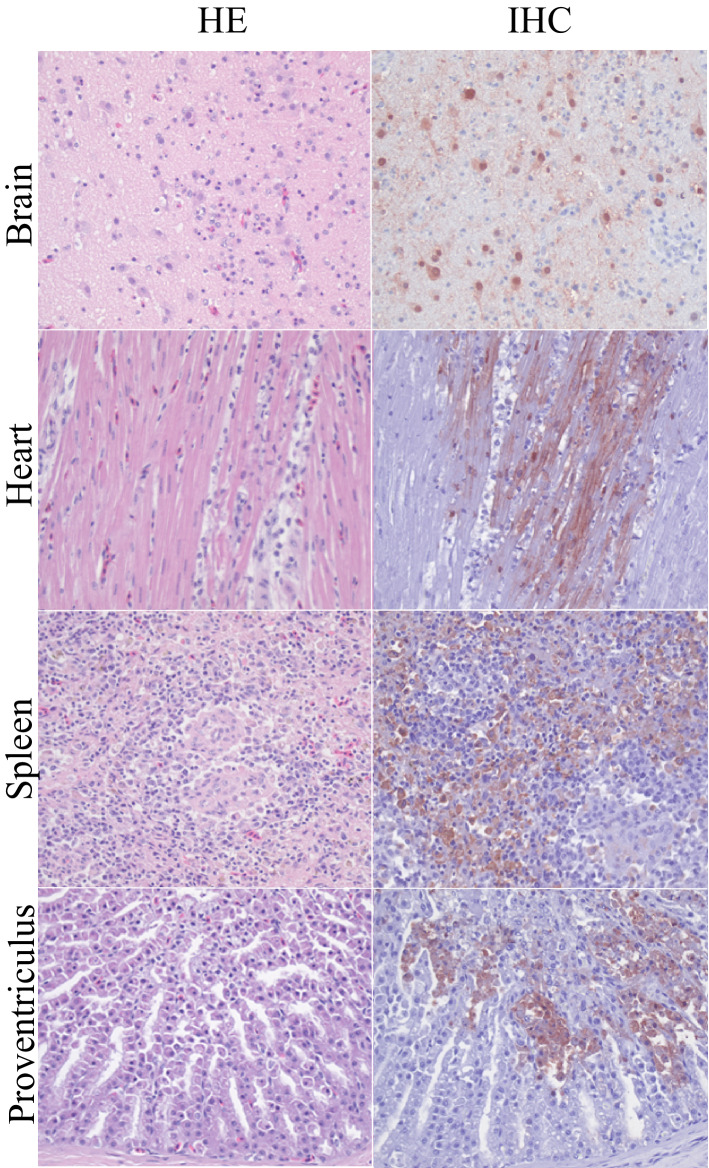
Histopathological changes and influenza virus antigen expression in tissues of HPAI H5N8 virus-infected Common buzzards (*Buteo buteo*). Tissue sections in the left column are stained with hematoxylin and eosin (HE). Serial tissue sections in the right column are stained for influenza virus antigen by immunohistochemistry (IHC). In all tissues there is necrosis and inflammation associated with virus antigen expression.

Several organs, and in particular the brain, heart, liver, and spleen, showed histological lesions that colocalized with moderate to abundant presence of viral antigen (Fig. 1). The brain of 81% (9/11) of the birds showed multifocal encephalitis with foci of gliosis, neuronal degeneration, and necrosis; abundant viral antigen was present in the nucleus and cytoplasm of several neurons. The heart of 63% (7/11) of the birds showed marked, multifocal to focally extensive myocardial necrosis; few myocardial cells presented viral antigen. The liver and the spleen of 54% (6/11) of the birds showed multifocal, mild to moderate hepatocellular and splenic necrosis, respectively; viral antigen was associated with areas of necrosis. The kidneys of 18% (2/11) of the birds showed mild interstitial nephritis, with no presence of viral antigen. The pancreas of 18% (2/11) of the birds showed multifocal necrosis with no presence of viral antigen. The ovary of 18% (2/11) of the birds showed moderate infiltration with mononuclear cells; few epithelial cells presented viral antigen. The proventriculus of 18% (2/11) of the birds showed necrotic lesions; few necrotic cells, most likely epithelial cells, presented viral antigen. Mild to moderate, inflammatory changes and infiltration with mononuclear cells were observed in the nasal passage, trachea, lung, and air sac of 18% (2/11) of the birds; few epithelial cells in these organs presented viral antigen.

## Discussion

This study describes the virological and pathological findings of Common buzzards infected with the 2020–2021 HPAI H5N8 virus. These analyses showed that the main lesions were HPAI virus-associated inflammation and necrosis in multiple tissues including brain and heart, confirming HPAI as cause of death or severe disease.

The Common buzzard presents with several characteristic traits that make it a valuable bioindicator of HPAIV presence in wildlife. It is a medium-sized raptor, present almost throughout Europe. In the Netherlands, its population has been stable since 1970 with an estimated maximum winter population of 30,000–50,000 individuals^16^. The Common buzzard is mainly a resident bird, which generally inhabits woodlands but is adaptable to wetlands^16,17^. Its feeding behavior as an opportunistic predator and scavenger has the potential to expose it to HPAIV-infected prey. Given these predisposing biological traits, it is not unexpected that Common buzzards accounted for the highest number of HPAI virus detections in raptors during the 2020–2021 epizootic.

Previous studies showed that HPAI viruses in raptors are highly neurotropic and cause severe neurological disease^8,10,15,18,19^. This study also supports those findings, as the most consistent lesion in Common buzzards was viral encephalitis, with confirmed presence of viral antigen in affected neurons. In addition to the nervous system, all the tissues tested of the Common buzzards were positive for virus based on RT-PCR and showed infection-related, histological lesions, indicating that HPAI H5N8 virus infection in the Common buzzard causes systemic disease.

This study showed that HPAI H5N8 virus is also highly cardiotropic, as the myocardium of the Common buzzards contained the highest amount of virus based on RT-PCR (Table 1), and virus-associated, severe histological lesions in 63% (7/11) birds. In addition, 54% (6/11) of the Common buzzards showed virus-associated lesions in the liver and spleen.

The Common buzzard is considered to be infected via the oral route by ingesting HPAIV-infected preys. Transmission of HPAIV from ingesting infected chicken meat has been experimentally confirmed in raptors^20^. Interestingly, the proventriculus of two birds in our study showed necrotic lesions with viral antigen. This finding further supports the oral route of infection, although we cannot exclude the possibility that the proventriculus was infected via the hematogenous route. It also provides new records of HPAIV enterotropism in wild birds. The adaptation to the intestinal tract is a mechanism recently reported for HPAI H5N8 virus, that may allow a more efficient fecal–oral transmission in wild birds^5^.

Real time PCR (RT-PCR) is the preferred test for HPAI virus detection for active and passive bird surveillance^9^. In this study, cloacal and pharyngeal swabs had comparable RNA-levels, and both were adequate for the detection of the virus. The tissue analysis by RT-PCR showed that heart, brain, and air sac had highest viral RNA concentrations compared to other organs. Although not confirmed by a quantitative real time PCR, the results obtained by RT-PCR are well supported by histopathology and immunohistochemistry. Our advice for diagnostic pathologists is to collect at least a miniset of samples including brain, heart, liver and spleen, as these tissues are relatively easily sampled and were positive by both RT-PCR and for virus-antigen-associated lesions. For virus diagnosis of Common buzzards found dead (but without the interest or possibility to perform pathological examination), it is enough to collect pharyngeal and cloacal swabs, because they were positive by RT-PCR with Ct values that were comparable to those in most tissues (with exception of heart, that had higher Ct values).

We did not detect antibodies against avian influenza virus NP in the sera of the Common buzzards in this study. Most of the birds (8/11) were juveniles in their first year of life, and likely they did not have protective antibodies from previous infections, as this was the first time in their lives that they experienced a HPAI epizootic. The absence of antibodies indicates also that the Common buzzards died acutely soon after infection, similarly to experimentally infected raptors that did not seroconvert before early death^19^. All the birds in our study were females. Females are larger than males (adult female weigh about 15% more than adult males), thus it is possible that female raptors are easier to find during surveillance or that there are sex-associated differences in feeding patterns.

This study showed that HPAIV infection in Common buzzards produced severe systemic disease, and subsequent acute death based on the stage of the pathological changes and absence of serum antibodies. Cloacal and pharyngeal swabs were comparable in detecting the infection. Many organs contained viral RNA; with heart, brain and air sac containing the highest amount of viral RNA. The proventriculus of two birds showed virus-associated lesions, implying a possible adaptation of the virus to the gastro-intestinal tract.

## Materials and methods

### Birds

In November 2020, during the first peak of the 2020–2021 HPAIV epizootic, 11 wild Common buzzard carcasses were presented at Erasmus MC, Rotterdam, for pathological and virological investigations. These birds were found by citizen scientists; nine birds were found dead, and two were still alive but subsequentially were euthanized after showing severe neurological signs of disease, such as torticollis and body tremors. The carcasses were refrigerated, and autopsies were performed at Bio Security Level 3 settings within 24 h after retrieval.

### Necropsy

The birds appeared freshly dead and in good state of preservation. All the birds were female based on the presence of an oviduct and ovaries in the coelomic cavity; eight were juveniles (first year) and three were adults (second year) based on their plumage. Pharyngeal and cloacal swabs were collected for virology from each bird, using sterile cotton swabs placed in 1 ml of virus transport medium. Tissue samples from nasal turbinate, air sac, trachea, lung, heart, liver, spleen, kidney, pancreas, stomach, jejunum, duodenum, colon, and brain were collected for virology and kept at -80 °C until analysis. Duplicate samples of the same tissue were collected for histopathology and immunohistochemistry and fixed in 10% neutral-buffered formalin until analysis. Blood from the heart was collected in a plain 2 ml tube, and centrifuged for 15 min at 1500 g.

Serology. Sera were tested for nucleoprotein (NP)-specific antibodies with a commercial blocking enzyme-linked immunosorbent assay (bELISA) (Idexx A Ab Test ; Idexx Laboratories BV, Hoofddorp, The Netherlands). Samples were tested according to the manufacturer’s instructions. A sample was considered NP positive when the signal-to-noise ratio (i.e., ratio of the mean optical density [ODx] of the sample/ODx of the negative control) was 0.5 or lower.

### Virology

Tissue samples were first weighed and then homogenized with a FastPrep 24 (MP Biomedicals, Eindhoven, The Netherlands) in Hankʾs balanced salt solution and centrifuged briefly before dilution in lysis buffer for RNA extraction^10^. Extracted total RNA of tissue samples, pharyngeal and cloacal swabs were tested for the presence of influenza A virus matrix gene-fragment; pharyngeal and cloacal swabs were further subtyped using real-time RT-PCR targeting fragments of the H5 and N8 genes. Samples were characterized as HPAI H5 virus by detection of a multi-basic cleavage site upon Sanger sequencing of the HA gene^9^. Full genome sequence of the virus isolated from the pharyngeal swab of Common buzzard 9 (B9), was obtained by Sanger sequencing. In order to assess the excretion of infectious virus, pharyngeal and cloacal swabs were also tested by virus culture. Virus titers of the pharyngeal and cloacal swabs were obtained with triplicate tenfold serial dilutions in confluent layers of Madin-Darby canine kidney (MDCK) cells^10^.

### Histopathology and immunohistochemistry

Tissues samples were embedded in paraffin, sectioned at 4 μm, and stained: with hematoxylin and eosin (HE) for presence of histopathological changes; with an immunohistochemical test, using monoclonal antibody against nucleoprotein of influenza A virus as a primary antibody, for detection of influenza viral antigen^10^.
